# Predictive cues elicit a liminal confirmation bias in the moral evaluation of real-world images

**DOI:** 10.3389/fpsyg.2024.1329116

**Published:** 2024-02-15

**Authors:** Chunyu Ma, Johan Lauwereyns

**Affiliations:** ^1^Graduate School of Systems Life Sciences, Kyushu University, Fukuoka, Japan; ^2^School of Interdisciplinary Science and Innovation, Kyushu University, Fukuoka, Japan; ^3^Faculty of Arts and Science, Kyushu University, Fukuoka, Japan

**Keywords:** confirmation bias, predictive cues, prior processing, moral image evaluation, liminal influence

## Abstract

Previous research suggested that predictive cues enhance the preference and reduce the response time for congruent targets during bivalent food evaluation, indicating a confirmation bias. Less is known about how prior processing affects subjective moral evaluation. Here, we used three different types of predictive cues to elicit directional vs. non-predictive prior processing and then asked the participants to perform moral evaluations on a continuous scale from −10 (“very immoral”) to +10 (“very moral”) with a diverse set of real-world images. Our experimental image database balanced the morality of image content and the volatility of the ratings based on the means and standard deviations in a preliminary study. Ratings, response times, and gaze positions were measured to examine the effects of predictive cues on the moral evaluation of real-world images. We found that the moral ratings were in line with the expectations induced by the cues. Compared to the non-predictive condition, the moral evaluation in the directional conditions was more polarized. For neutral images, the predictive cues tilted the evaluations to positive vs. negative, indicating a decisive liminal influence. High-volatility images were impacted more than low-volatility images in ratings as well as response times. Furthermore, the gaze positions during the interval between the predictive cue and the image showed a spatial displacement in line with the cue instruction, indicating a response bias. Together, the results show that predictive cues elicit a liminal confirmation bias in moral image evaluation, much in the same way as in bivalent food evaluation.

## Introduction

Confirmation bias is a well-established phenomenon in the field of psychology, referring to the tendency for individuals to look for the presence of what they expect, or to favor information that supports their existing beliefs or opinions, while disregarding information that contradicts them (Klayman, 1995; Oswald and Grosjean, 2004). It has been demonstrated in various domains and discussed widely on its utility and disutility (Nickerson, 1998). For instance, confirmation biases in political beliefs can lead to attitude polarization, which can hinder the development of rational behavior in a democracy (Taber and Lodge, 2006); as well as in economics (Koriat et al., 2006; Bisière et al., 2015; Charness and Dave, 2017), science (Kahan et al., 2011), and medicine (Mendel et al., 2011; Kassin et al., 2013). Moreover, Rajsic et al. (2015, 2018) conducted a series of experiments that proved people's pre-existing beliefs can influence their perception and decision-making in visual search tasks.

With respect to subjective evaluation, previous research in our laboratory (Ounjai et al., 2018) showed that bivalent predictive cues have a large impact on preference ratings for food images. Correct predictions of appetitive or aversive food images produced quick and amplified evaluation scores, while erroneous predictions elicited slower and less extreme evaluation scores. The data from gaze positioning further suggested that the predictive cues produced anticipatory biases in the cued evaluation direction. The entire pattern of data suggested the operation of an active confirmation bias. In the follow-up study (Ounjai et al., 2020) we found that the predictive cues were most influential for food images that overall had relatively moderate rating scores. This finding suggested that the confirmation bias has a specific liminal influence, able to nudge the evaluation in a positive or negative direction particularly for stimuli near the boundary of neutrality. Here, we aim to further investigate this “liminal confirmation bias,” defined as a confirmation bias that operates specifically near the boundary of neutrality in a bivalent evaluation system.

In the present study, we opted to investigate the construct of liminal confirmation bias with a completely different type of human judgment, namely moral evaluation. In doing so, we envisaged a conceptual replication that would not only increase our understanding of confirmatory mechanisms in human judgment, but also make an explicit connection to the study of moral cognition (see Levari et al., 2018; for a similar approach to studying core mechanisms of human judgment).

It should be noted that the study of moral cognition is highly complex with the separable dimensions of decision-making, judgment, and inference (Yu et al., 2019; Ponsi et al., 2021). Here we focus on moral judgment as the process of evaluating events or behaviors that have a moral component. Moral judgment involves considering personal values, ethical principles, emotional associations, and societal norms (Forsyth, 1980; Turiel, 1983; Greene and Haidt, 2002; Bechara, 2004; Lee and Gino, 2015; Jung et al., 2016).

While some moral evaluations are straightforward, eliciting strong and unambiguous responses (Haidt, 2001), others are difficult to judge, leading to hesitation and multiple viewpoints as seen in the case of dilemmas (Greene et al., 2001; Rom and Conway, 2018). Confirmation bias may have a distinct impact depending on the type of processing for moral evaluation. One widely held theory suggests that confirmation bias is most pronounced in the case of deeply ingrained, ideological, or emotionally charged views (Nickerson, 1998; Vedejová and Cavojová, 2022).

Other researchers argued that confirmation bias particularly takes effect on evaluations with respect to more ambiguous content. Klayman and Ha (1987) found that people tend to seek out information that confirms their initial beliefs when the hypotheses are more complex, contentious, or difficult to evaluate.

However, the extent to which confirmation bias influences moral evaluation remains largely unknown. This study aims to investigate how confirmation bias affects moral evaluations using bivalent predictive cues vs. non-predictive cues. We hypothesize that, in line with our prior research on preference judgment using bivalent food images, predictive cues will elicit a congruent bias on moral evaluation such that the ratings align with the predictions. Here, the predictive cues were followed by congruent images in two-thirds of the trials (i.e., moral images after positive predictions; immoral images after negative predictions) or by neutral images in one-third of the trials. Non-predictive cues (in the shape of gray question marks) were followed equally likely by moral, immoral, or neutral images. We expected the ratings of the images to align with the predictions. For neutral images, we expected to observe a liminal confirmation bias such that the ratings would be tilted in the direction indicated by the predictive cues. The response times following predictive cues would be shorter than following non-predictive cues. The gaze positions during the interval between the predictive cue and the real-world image should show a spatial displacement in line with the prediction (i.e., positive predictive cues will lead to a gaze bias toward the positive side of the rating scale and vice versa).

To examine our hypotheses, we opted to use a well-established database of socio-moral images (SMID; Crone et al., 2018) to select a stimulus set that balanced the morality of image content and the volatility of the ratings based on the means and standard deviations in a preliminary study. In our within-subjects design, image type (moral, neutral, immoral), volatility (high-volatility, low-volatility), and predictive cue (directional, non-predictive) were explored as the main factors for confirmation bias in the moral evaluation of real-world images. The study was preregistered at OSF (https://osf.io/pe36b).

## Methods

### Participants

A total of *N* = 30 individuals at Kyushu University (17 males and 13 females with a mean age of 20.83 years old, and a standard deviation of 2.93) participated in the study. All participants completed the entire experiment, and their physiological responses were successfully captured by the eye tracker and biometric kit. The participants had no previous experience in any similar experiment. No participant reported any vision or health issue, or past or present psychological disorder. All participants had normal or corrected-to-normal vision. The Human Ethics Committee of the Faculty of Arts and Science at Kyushu University approved the study. Participants were asked to give their informed consent in writing and fill out a pre-questionnaire prior to the experiment; the informed consent included a no-risk statement and rights protection. Each participant received 1,000 yen per hour as monetary compensation for their participation.

### Stimuli and preliminary study

To prepare the stimulus set for the present study, we proceeded in three steps. First, we preselected 620 images from SMID. Then, using these images we conducted a rating experiment to create a bivalent evaluation system with a sample of 14 participants at Kyushu University. Finally, we selected a balanced stimulus set by combining the bivalent evaluation data with the data of standard deviations from SMID. Detailed information on this procedure is provided in the Supplementary material.

**Figure 1:**
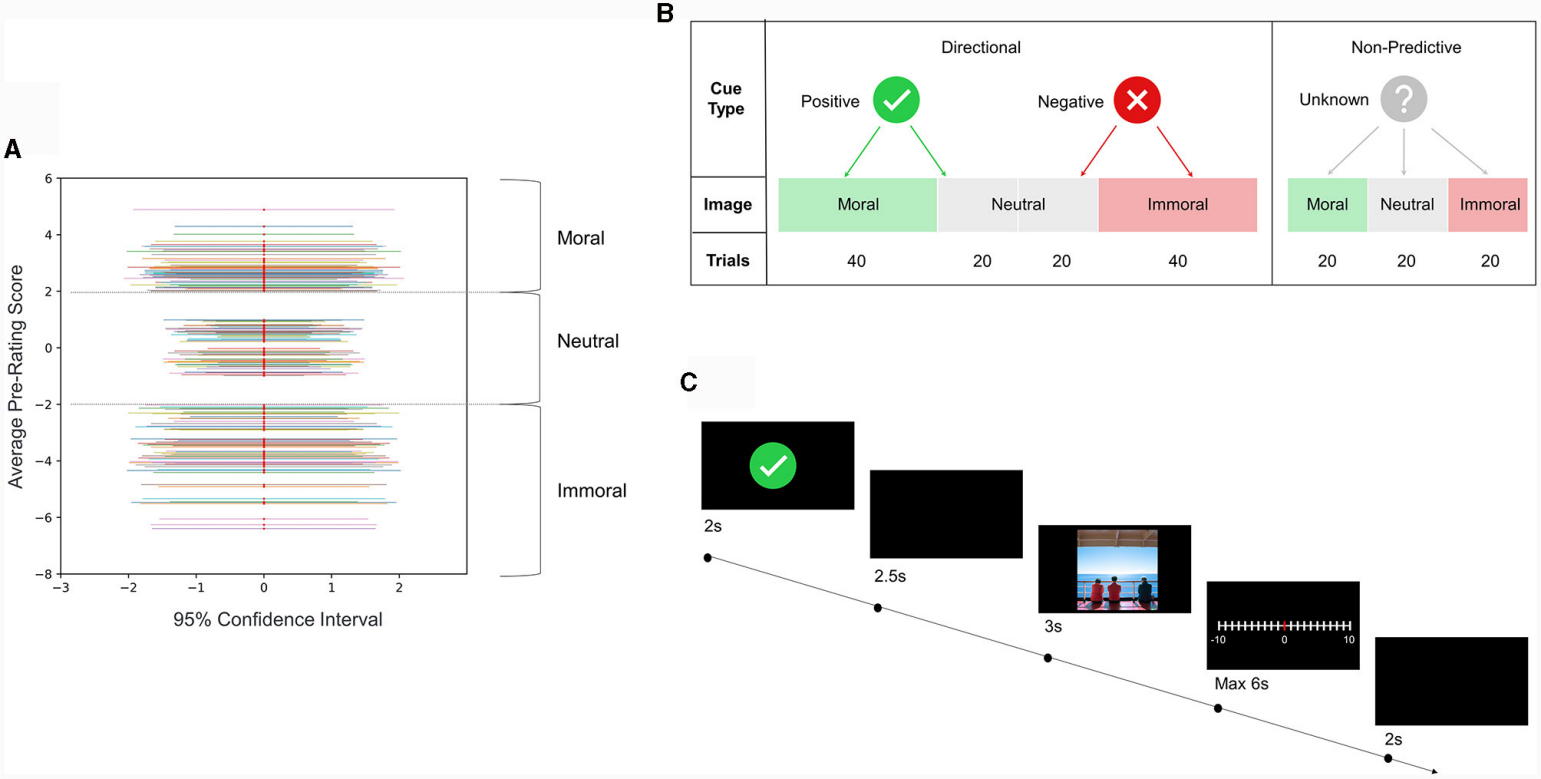
**(A)** Distribution, volatility, and classification of the experimental image database. The horizontal axis presents the 95% Confidence Interval (CI) of the pre-rating score, and the red dot represents the average pre-rating moral mean for each image. Based on the prior moral evaluations, dashed lines are used to divide the range of moral content into three categories: moral, neutral, and immoral. The entire dataset of moral images was evenly distributed among these three categories. **(B)** The experimental design consisted of all possible experimental conditions and corresponding trial quantities in the moral evaluation task. Directional cues and non-predictive cue were paired with moral, neutral, and immoral images. Two types of directional cues, namely positive and negative cues, were used to introduce valid outcomes vs. neutral outcomes in a fixed ratio of 2:1, ensuring the effectiveness of the directional cue. Additionally, one type of non-predictive cue, referred to as the non-predictive cue, was paired with images from all three categories in equal ratios, thus eliminating any directional effects. **(C)** The schematic diagram gives an example of the experimental procedure with a positive-cue trial. Each trial has 5 frames: 2s-cue, 2.5s-delay, 3s-stimuli, Self-paced evaluation (no more than 6s), and 2s-ITI. Participants were provided with a joystick that allowed them to adjust their ratings of immorality or morality on a scale ranging from −10 to +10 by bending in the left or right direction. The initial position of the joystick was set at 0. The non-copyrighted image shown in this example is used only for illustrative purposes and was not part of the actual image database.

The final distribution of moral content and volatility of the image set is shown in Figure 1A. Based on the average pre-rating scores of the images, the three levels of morality were set, respectively, as “Moral” for a rating higher than 2, “Neutral” for a rating between −1 and 1, and “Immoral” for a rating lower than −2; also in this set, the number of high-volatility images (high standard deviation in SMID, SD > 1) equals the number of low-volatility images (low standard deviation in SMID, SD < 1) at the corresponding morality level.

### Apparatus

The experiment was run by PsychoPy 3 (version 2021.2.3) connecting with a joystick, an eye tracker (GazePoint GP3, sampling rate of 150 Hz) and a biometric kit (to record GSR and heart rate). All stimuli were presented on a DELL 23.8-inch full high-definition flat-panel monitor with a resolution of 1,920 by 1,080 pixels and a refresh rate of 60 Hz. A chinrest was mounted at 60 cm from the eye tracker and at 100 cm from the monitor to fix the head location.

### Moral evaluation task

The conditions for the moral evaluation task consisted of seven types with three levels of prediction (Positive, Negative, and Unknown) and three levels of morality of the image content (Moral, Neutral, and Immoral) in a total of 180 trials. The experimental design is shown in Figure 1B. Directional cues (including Positive and Negative) were used to indicate a valid prediction before stimuli exposure in two-thirds of the trials, while in the remaining third of the trials, the directional cues were followed by neutral images. As for non-predictive cues (Unknown), the content of the following image was equally likely to be Moral, Neutral, or Immoral.

Participants were introduced to the trial structure illustrated in Figure 1C and the joystick manipulation method to ensure they were familiar with the evaluation task. All trials started with a 2 s cue at the center of the display. Then, a 2.5 s delay with a blank screen was presented before the target image onset. The target image was displayed for 3 s at the center of the screen. Then the participants had to rate the depicted content on a continuous scale from −10 (“very immoral”) to +10 (“very moral”) by bending the joystick in the corresponding direction and clicking the trigger on the joystick to mark the evaluation. The rating had to be completed within 6 s in each trial, or the trial would be aborted and recorded as missing data. At the end of each trial, there was an inter-trial-interval (ITI) of 2 s, with a blank screen.

Before the actual data collection, participants were given detailed task instructions and presented with 10 practice trials to familiarize themselves with the tasks and the operation of the joystick. It was clearly explained that predictive cues were 100% reliable whereas non-predictive cues had no predictive value. No image was presented more than once. The assignment of images to directional vs. non-predictive conditions was counterbalanced across participants. All trials were presented in a randomized order for each participant.

## Results

We conducted a power analysis using the software program G^*^Power to set the appropriate sample size beforehand. The goal was to obtain 0.95 power to detect a medium effect size of 0.25 at the standard 0.05 alpha error probability. In accordance with the targeted sample size, 30 participants were recruited and completed the entire experiment. All participants completed all trials that provided sufficient data for the present analyses, and their physiological responses were successfully captured by the eye tracker.

### Manual response analysis

**Figure 2:**
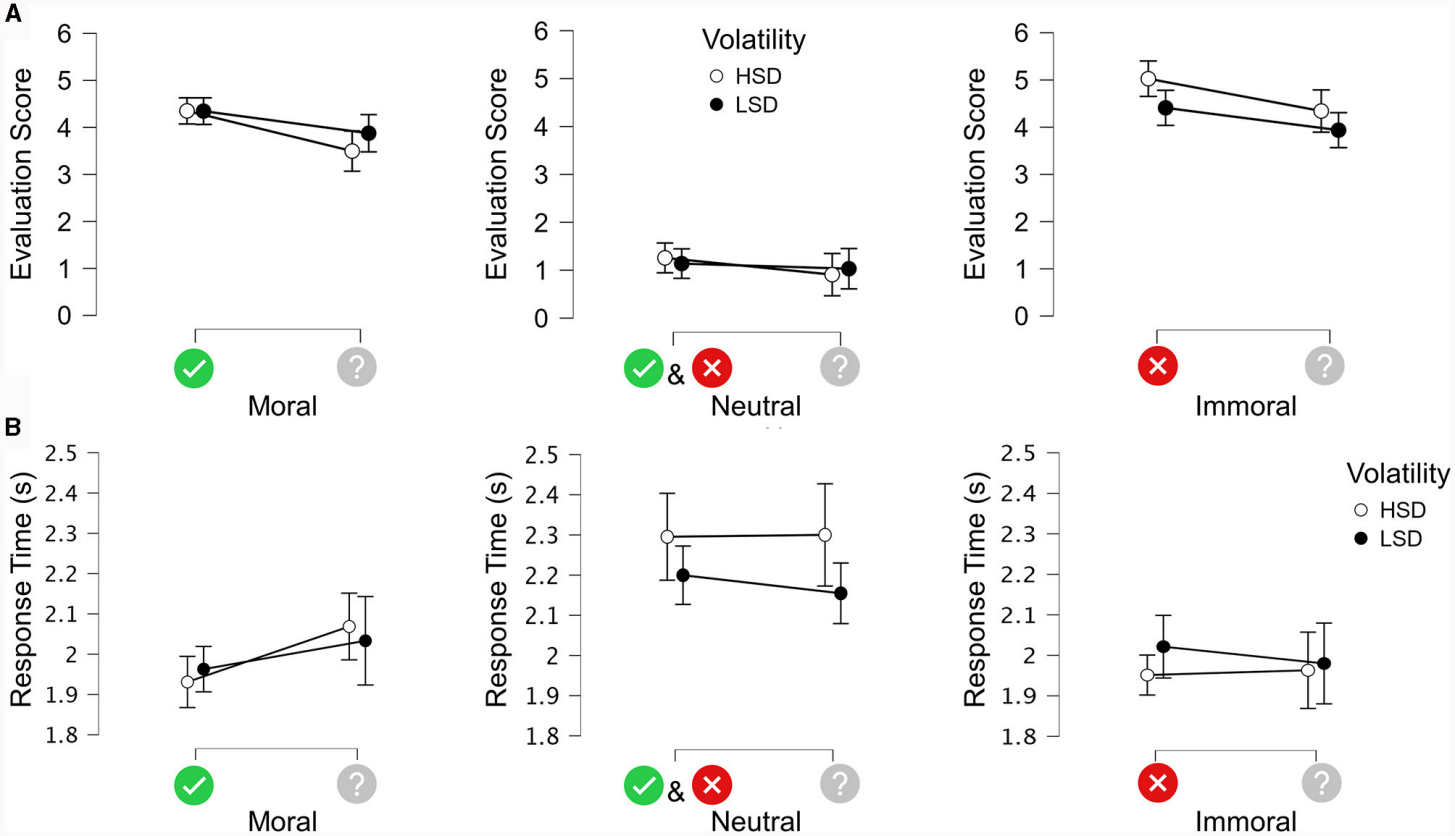
**(A)** Average evaluation scores for each level of morality of the image content in the directional vs. non-predictive condition grouped by volatility split into HSD and LSD. The error bars indicate 95% CI. **(B)** Average response times for each level of morality of the image content in directional vs. non-predictive condition grouped by volatility split into HSD and LSD. The error bars indicate 95% CI.

**Figure 3:**
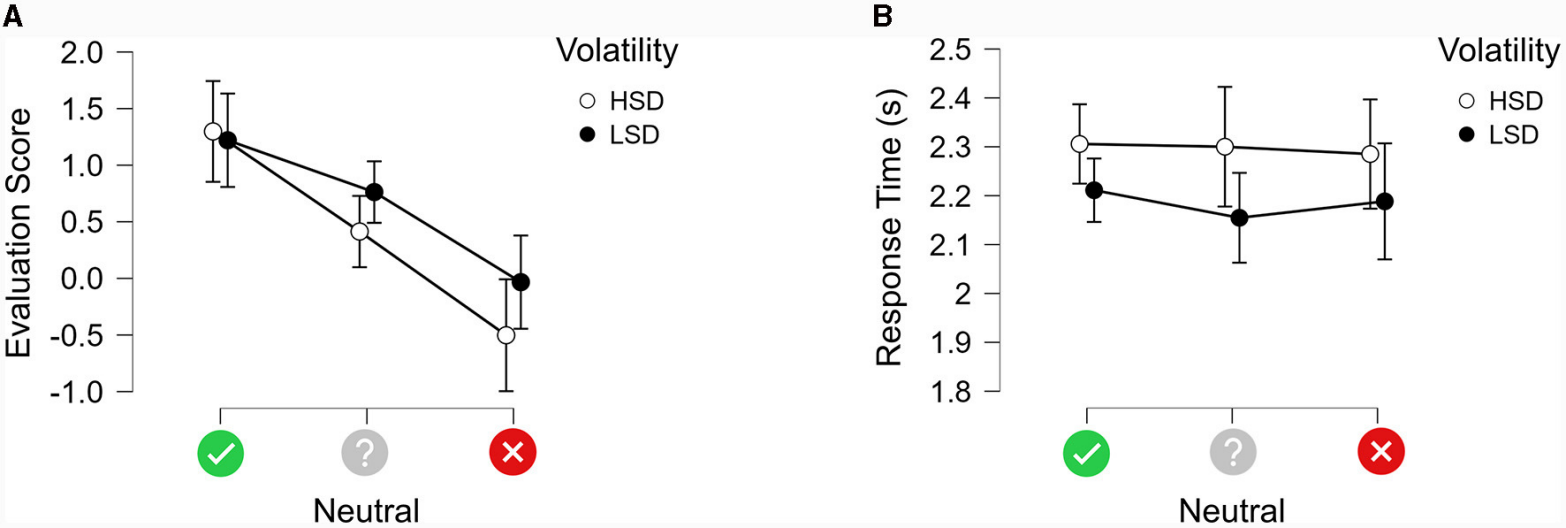
**(A)** Average evaluation scores for neutral images following the three types of cues grouped by volatility split into HSD and LSD. The error bars indicate 95% CI. **(B)** Average response times for neutral images following the three types of cues grouped by volatility split into HSD and LSD. The error bars indicate 95% CI.

We focused on the evaluation scores and response times as dependent measures in the manual responses. The evaluation scores were defined as the ratings on a scale from −10 (“very immoral”) to +10 (“very moral”), and the response times were defined as the time from the onset of the scale to the click on the trigger of the joystick. In order to compare cue effects for directional cues vs. non-predictive cues across conditions, we took the absolute values of the evaluation scores for all types of images (Figure 2). In this analysis, the objective was to check whether predictive cues elicited more extreme scores than non-predictive cues. When comparing the cue effects for neutral images (Figure 3) across positive direction cues, negative direction cues, and non-predictive cues, we kept the signs of the evaluation scores to check whether the direction of prediction could polarize the evaluation of neutral images. We opted to conduct repeated measure ANOVA to analyze the cue effects for each type of morality content using the JASP software package. Conventional p statistics results were assessed to show significant main effects or interaction effects regarding factors with more than two levels, with the error bars representing the 95% confidence intervals around the means.

### Directional cues vs. non-predictive cues

Figure 2A presents the means and 95% confidence intervals (CI) of the evaluation scores for each volatility (HSD and LSD) as a function of the type of cue (directional or non-predictive) and the morality of the image content, which was moral (left panel), neutral (middle panel), or immoral (right panel). To assess the effects statistically, we conducted a 2 × 3 × 2 repeated measure ANOVA with the within-subject factors Cue Type (directional vs. non-predictive), Image Content (moral, neutral, or immoral) and Volatility of the evaluation on the image content (high vs. low). The results produced a significant main effect of Image Content [F_(2, 58)_ = 126.214, MSE = 3.165, η^2^ *p* = 0.813, *p* < 0.001]. *Post-hoc* comparisons using Bonferroni correction showed that neutral images obtained significantly lower scores on average (1.084) than both immoral images (4.429, *p* < 0.001) and moral images (4.018, *p* < 0.001), whereas there was no significant difference between moral and immoral images. There was also a significant main effect of Cue Type [F_(1, 29)_ = 17.204, MSE = 1.256, η^2^*p* = 0.372, *p* < 0.001], with directional cues producing higher scores on average (3.422) than non-predictive cues (2.932). No significant main effect of Volatility was found [F_(1, 29)_ = 2.528, MSE = 0.409, *p* = 0.123]. There were significant interactions between Image Content and Cue Type [F_(2, 58)_ =3.214, MSE = 0.496, η^2^*p* = 0.100, *p* < 0.05]; between Image Content and Volatility [F_(2, 58)_ =10.507, MSE = 0.374, η^2^*p* = 0.266, *p* < 0.001); and between Cue Type and Volatility [F_(1, 29)_ = 9.781, MSE = 0.180, η^2^*p* = 0.252, *p* = *p* < 0.05). The three-way interaction was not significant [F_(2, 58)_ =0.115, MSE = 0.557, *p* = 0.892]. *Post-hoc* comparisons for the significant interactions are reported in detail in the Supplementary material.

Figure 2B shows the means and 95% CI of the response times for directional vs. non-predictive cues for each volatility as a function of the morality of the image content. The three-way repeated measure ANOVA produced a significant main effect of Image Content [F_(2, 58)_ = 27.412, MSE = 0.091, η^2^*p* = 0.486, *p* < 0.001]. *Post-hoc* comparisons using Bonferroni correction showed that neutral images produced significantly slower response times (2.238 s) than moral images (1.999 s, *p* < 0.001) and immoral images (1.979 s, *p* < 0.001), whereas there was no significant difference between response times for moral vs. immoral images. The main effect of Cue Type was not significant [F_(1, 29)_ = 0.567, MSE = 0.084, *p* = 0.457], nor was the main effect of Volatility [F_(1, 29)_ = 3.228, MSE = 0.019, *p* = 0.083]. There was a significant interaction between Image Content and Cue Type [F_(2, 58)_ = 3.193, MSE = 0.046, η^2^*p* = 0.099, *p* < 0.05]. There was also a significant interaction between Image Content and Volatility [F_(2, 58)_ = 3.754, MSE = 0.057, η^2^*p* = 0.115, *p* < 0.05], but not between Volatility and Cue Type [F_(2, 58)_ = 1.432, MSE = 0.050, *p* = 0.241]. The three-way interaction was not significant [F_(2, 58)_ = 0.020, MSE = 0.032, *p* = 0.980]. *Post-hoc* comparisons for the significant interactions are reported in detail in the Supplementary material.

### Cue effects for neutral images

In order to separate out how the different cues impacted on the ratings of neutral images, we analyzed the signed evaluation scores of high and low-volatility neutral images for the three types of cues, as shown in Figure 3A. The 3 × 2 repeated measures ANOVA produced a significant main effect of Cue type [F_(2, 58)_ = 18.040, MSE = 1.948, η^2^*p* = 0.384, *p* < 0.001]. *Post-hoc* comparisons using Bonferroni correction showed that positive predictions produced higher ratings (1.259) than non-predictive cues (0.588, *p* < 0.05) and negative predictions (−0.256, *p* < 0.001); negative predictions also produced significantly lower ratings than non-predictive cues (*p* < 0.005). There was a significant main effect of Volatility [F_(1, 29)_ = 4.605, MSE = 0.595, η^2^*p* = 0.137, *p* < 0.05], with high-volatility images producing lower ratings (0.404) than low-volatility images (0.649). There was no significant interaction between Cue Type and Volatility [F_(2, 58)_ = 2.035, MSE = 0.609, *p* = 0.140].

Figure 3B presents the response times for high and low volatility as a function of each type of cue. The 3 × 2 repeated measures ANOVA indicated that Volatility had a significant main effect [F_(1, 29)_ = 6.646, MSE = 0.085, η^2^*p* = 0.186, *p* = 0.015], confirming that high-volatility neutral images (2.297 s) took longer to rate than low-volatility neutral images (2.185 s). There was no significant main effect of Cue Type [F_(2, 58)_ = 0.173, MSE = 0.088, *p* = 0.842), nor was there a significant interaction between Volatility and Cue Type [F_(2, 58)_ = 0.242, MSE = 0.051, *p* = 0.786].

### Gaze displacement analysis

To examine the hypothesis that the predictive cues would elicit a response bias, we analyzed the average gaze position during the delay period (2.5 s). An active response bias would be visualized by the participant's gaze displacement along the direction associated with the predicted outcome (immoral–left, moral–right). This gaze positioning analysis focused on the period from the disappearance of the cue until the target onset. The data were classified into three conditions according to the Cue Type (positive, non-predictive, and negative).

**Figure 4:**
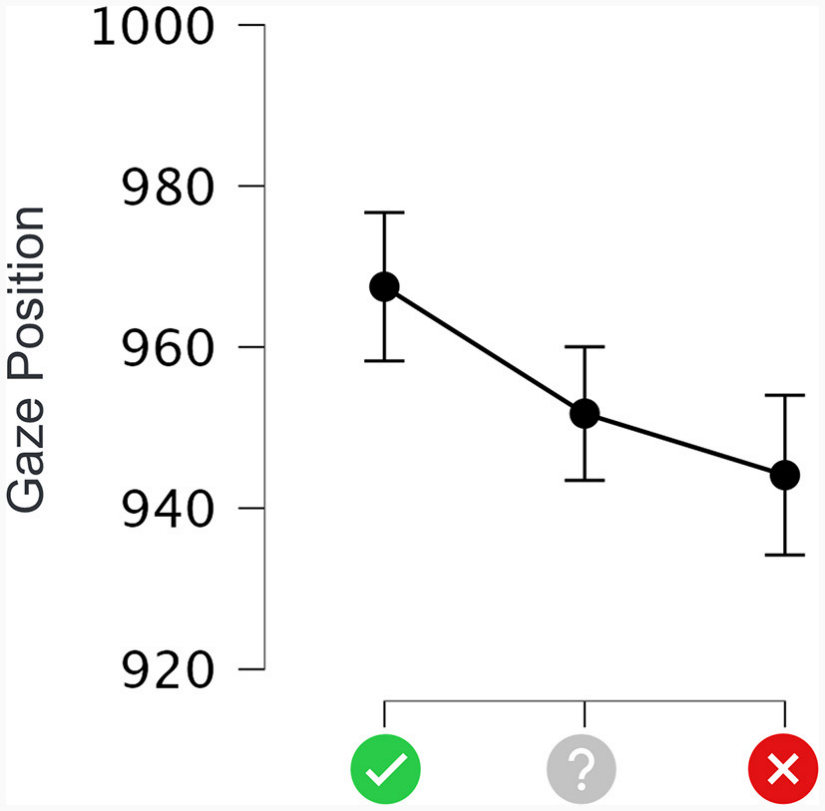
Average horizontal gaze positions during the delay period as a function of cue type. The X-axis shows each type of cue. The Y-axis represents the horizontal position on the screen, ranging from 0 to 1,920 pixels from left to right. The error bars indicate 95% CI.

Figure 4 presents the average horizontal gaze positions during the delay as a function of Cue Type. Our display frame had a width of 1,920 pix starting from the left edge, and the cue was presented at the center of the screen with a horizontal coordinate at 960 pix. A one-way repeated measures ANOVA showed that Cue Type produced a significant main effect on gaze position [F_(2, 58)_ = 7.086, MSE = 602.422, *p* < 0.05]. The average horizontal gaze positions shifted to the left or right in the direction congruent with the cue type. *Post hoc* comparisons with Bonferroni correction showed that positive predictive cues produced more rightward gaze positions (967.496 pix) than non-predictive cues (951.744 pix; *p* < 0.05) and negative predictive cues (944.102 pix; *p* < 0.001). The difference between non-predictive and negative predictive cues was not significant.

## Discussion

In the present study, we aimed to investigate whether the confirmation bias influences moral evaluations of real-world images and, if so, under which conditions this bias operates. To this end, we utilized a diverse set of images taken from SMID spanning the moral spectrum (ranging from “moral” to “neutral” to “immoral”) and volatility (with a small or large standard deviation in ratings). We asked participants to evaluate these images and rate their evaluations on a continuous scale from +10 to −10.

The current results demonstrate that the moral ratings were in line with the expectations induced by the cue, and that the neutral images required longer deliberation times to make an evaluation than moral or immoral images. We found that the average evaluation score after directional instruction was higher than after non-predictive instruction, especially when evaluating moral images and immoral images, while the response times showed no difference. These results indicated that the Cue Type worked on the moral evaluation procedure by shifting the perceived degree of morality. Moreover, the Cue Type effects on the ratings of high-volatility images were more significant than on low-volatility images. Volatility further produced a significant main effect on response time, with shorter response times for low-volatility images than for high-volatility images. It is most likely that, for high-volatility images, subjects had to spend more effort to process the relation between cue and image to make their evaluation.

Our analysis revealed that directional cues had a substantial effect on images that were clearly moral or immoral, leading to more extreme evaluations due to confirmation bias, corroborating our previous observations of confirmation bias in the evaluation of appetitive and aversive food images (Ounjai et al., 2018, 2020). To examine the operation of a liminal confirmation bias in moral evaluation, we investigated the impact of predictive cues on the evaluation of neutral images which imply a different and more complex evaluation of moral properties. Both positive and negative predictive cues had a marked impact on the moral evaluation score, proving that participants referred to prior expectations in the moral evaluation of the neutral images. To our knowledge, the present findings are the first in the literature on moral cognition to track a decisive liminal influence of confirmation bias, effectively nudging the evaluation of neutral images to the positive or negative direction as a function of the predictive cue.

The gaze positions during the interval between the predictive cue and the image to be rated showed that the positive predictive cues led to a gaze bias toward the positive side of the rating scale, and negative predictive cues had the opposite effect. These spatial displacements reflected the prediction-forming process and were consistent with our previous study on preference decision-making using food images (Ounjai et al., 2018). Future studies could investigate the physiological responses involved in forming the response bias while controlling for individual differences, illumination (Proulx and Egeth, 2006) and color of the cue appearance.

The present effects of confirmation bias in moral evaluation, obtained with predictive cues on a range of real-world images, reflect the judgment processes given a particular level of reliability of the cues. Effectively, the directional predictions (with a check or cross symbol) were paired with moral or immoral images on two-thirds of the occasions, and with neutral images on the remaining third of the occasions. In this regime, the participants would effectively be reinforced into viewing the predictive cues as reliable. Given this perceived reliability, the participants gave more extreme ratings to moral and immoral images, in line with the prediction. These results are in line with the notion that confidence drives confirmation bias in simple decisions: People showed the strongest confirmation bias when they were already confident about the information to be confirmed (Rollwage et al., 2020). In other words, the predictive cues were validated, through experience, as pieces of information that warranted confirmation. Here, it is particularly relevant to note that this confirmation had a decisive impact on liminal cases, that is, neutral images.

The phenomenon of liminal confirmation bias may be structurally similar to that of the prevalence-induced concept change in human judgment (Levari et al., 2018), where ratings are influenced by the expectations set through prior experience. When participants expect half of the stimuli to be of a certain type, they adjust their criteria of categorization to meet that expectation. This implies shifts of judgment near the boundary of neutrality in a binary categorization system. Sometimes the exact same stimulus would be categorized differently depending on the base rate of occurrence. In the present paradigm, the expectation that predictive cues are valid leads the participants to adjust their criteria to the point that neutral images become polarized in line with the prediction.

This observation also raises the question how the base rates of expectation, or the history of reinforcement with the predictive cues, impact the dynamics of confirmation bias. The perceived reliability of the predictive cue may be differentially impacted by clear violations (e.g., a predictive cue followed by an image of the opposite polarity) than by neutral images. Also, there may be a limit to the number of neutral images that can be paired with predictive cues without diminishing the perceived reliability of the predictive cue.

More generally, the present findings of a liminal confirmation bias in the evaluation of real-world images have several important implications for the study of moral cognition. Theoretically, the observation that neutral images become polarized in the context of predictive cues may be connected to the logic of universalization in moral judgment (Levine et al., 2020). Indeed, the liminal confirmation bias functions structurally as a consensus mechanism that offers direction particularly in dilemmas or borderline cases when faced with a binary categorization task. In turn, this critical influence near the boundary of neutrality warrants further investigation to assess its effectiveness in real-world settings (Hertz et al., 2023). The notion that even simple, bivalent cues can serve to swing judgment from neutrality to being morally charged must be regarded as a dual-use type of knowledge that could promote or impede cooperative behavior (Capraro et al., 2019; Balafoutas and Rezaei, 2022). Thus, the present paradigm offers a critical tool for researchers to track the liminal influences of confirmation bias in moral judgment.

## Data availability statement

The datasets presented in this study can be found in online repositories. The names of the repository/repositories and accession number(s) can be found below: OSF Repository, https://doi.org/10.17605/OSF.IO/KXW7M.

## Ethics statement

The studies involving humans were approved by the Human Ethics Committee of the Faculty of Arts and Science at Kyushu University. The studies were conducted in accordance with the local legislation and institutional requirements. The participants provided their written informed consent to participate in this study.

## Author contributions

CM: Conceptualization, Data curation, Formal analysis, Investigation, Methodology, Software, Writing – original draft, Writing – review & editing. JL: Conceptualization, Funding acquisition, Resources, Supervision, Writing – original draft, Writing – review & editing.
