# Supplementary material for: Predictive cues elicit a liminal confirmation bias in the moral evaluation of real-world images
Source: Front Psychol. 2024 Feb 15;15:1329116. doi: 10.3389/fpsyg.2024.1329116 (PMC10902465; doi:10.3389/fpsyg.2024.1329116)
Supplement: Supplementary file 1 [file Data_Sheet_1.pdf]

Predictive Cues Elicit a Liminal Confirmation Bias in the Moral Evaluation of Real-World Images:

Supplementary Material

Chunyu Ma[1], Johan Lauwereyns[1,2,3*]

[1]Graduate School of Systems Life Sciences, Kyushu University, Fukuoka, Japan

[2]School of Interdisciplinary Science and Innovation, Kyushu University, Fukuoka, Japan

[3]Faculty of Arts and Science, Kyushu University, Fukuoka, Japan

*** Correspondence:**

Johan Lauwereyns

jl@artsci.kyushu-u.ac.jp

1. Supplementary Information to the Methods Section: Further Information on "Stimuli and Preliminary Study"

To prepare the stimulus set for the present study, we proceeded in three steps. First, we preselected 620 images from SMID. Then, using these images we conducted a rating experiment to create a bivalent evaluation system with a sample of 14 participants at Kyushu University. Finally, we selected a balanced stimulus set by combining the bivalent evaluation data with the data of standard deviations from SMID.

Preselection of 620 images from SMID

Based on the original categorization in the moral domain of SMID, we used code to randomly preselect 620 images from the entire database in an effort to prepare balanced preparatory sets of high and low standard deviation images for each category of Moral, Neutral, and Immoral (see Supplementary Figure 1).

Supplementary Figure 1

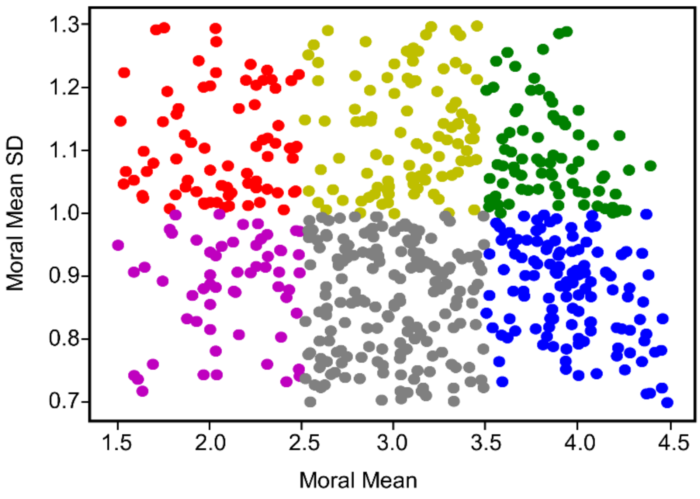

Supplementary Figure 1. Scatterplot of Moral Mean against Moral Mean Standard Deviation (SD) of 620 images selected from SMID used for the preparatory rating experiment. Using the mean moral rating in SMID, the stimulus set is divided into three levels: low (pink and red; moral mean < 2.5), middle (grey and yellow; moral mean between 2.5 and 3.5), and high (blue and green; moral mean > 3.5). Across the moral spectrum, the images elicited a wide range of variability in ratings, with some images eliciting nearly uniform judgments (with SDs well below 1), and others eliciting substantial variability (SDs of around 1.5). Since more than 98% of the images in SMID showed SDs ranging from 0.5 to 1.5, we used the average (SD = 1) to divide the stimulus set into two volatility categories, with “high volatility” (SD > 1) and “low volatility” (SD < 1). To balance the range of each morality and volatility category, we programmed code to select images that have a moral mean between 1.5 and 4.5, and an SD between 0.7 to 1.3. Specifically, we aimed for a balanced distribution of 30 stimuli in each of the 2 x 3 categories. Thus, red points represent immoral high volatility images; pink points represent immoral low volatility images; yellow points represent neutral high volatility images; gray points represent neutral low volatility images; green points represent moral high volatility images; and blue represents moral low volatility.

Creating a bivalent evaluation system with a participant sample at Kyushu University

In order to translate the ratings of SMID (ranging from 0 to 5) into a bivalent system with positive and negative poles, as well as to confirm whether the moral categories from SMID were reproducible with a group of participants from a different cultural background as compared to the SMID sample, we conducted a preparatory rating experiment with N = 14 students at the Laboratory of Cognitive Neuroscience of Kyushu University (8 males and 6 females with a mean age of 25.14 years old, and a standard deviation of 3.83). The

participants were asked to freely rate the preselected images from the SMID. The images were shown one by one, and the participants could bend the joystick to enter a rating frame; there was no time limitation for viewing or rating, but the participants could not return to the viewing frame once they had moved on to the rating frame. All images were adjusted keeping the original proportions to the same height at 810 pixels, equivalent to three-quarters of the screen (1920 × 1080 pixels), centering on the screen, with 96 dpi for horizontal and vertical resolution. Different from the rating rules of SMID, which ranged from 0-5, the images in our preliminary study were rated for bivalence with respect to moral content, from -10 (extremely immoral) to +10 (extremely moral). In this way, we obtained moral evaluations ranging from -10 to +10 for the 620 preselected SMID images using presentation conditions and working with a sample of participants similar to our main study.

Selecting a balanced stimulus set by combining the data

In the final step toward a balanced stimulus set, we used only images with good reproducibility (i.e., consistent categorization) across the SMID moral ratings and the ratings in our preliminary study. To make the final selections, for each image we used the moral ratings from our preliminary study and the corresponding SDs from the SMID. We randomly selected 180 real-world images by code, aiming for a balanced set with 60 moral images, 60 neutral images, and 60 immoral images. The final distribution of moral content and volatility of the image set is shown in Figure 1(A). Based on the average pre-rating scores of the images, the three levels of Image Content were set, respectively, as “Moral” for a rating higher than 2, “Neutral” for a rating between -1 and 1, and “Immoral” for a rating lower than -2; also in this set, the number of high-volatility images (high standard deviation in SMID, SD > 1) equals the number of low-volatility images (low standard deviation in SMID, SD < 1) at the corresponding morality level. Supplementary Table 1 presents the average moral

ratings and SDs as obtained in our preliminary study for each category; the data confirmed that the volatility level was also consistent across the SMID sample and that of our preliminary study.

Supplementary Table 1. The Average Moral Ratings and SDs for Each Category of Images, Based on the Preliminary Study.

	Moral-HSD	Moral-LSD	Neutral-HSD	Neutral-LSD	Immoral-HSD	Immoral-LSD
Valid	30	30	30	30	30	30
Average Rating	2.850	2.719	0.065	0.121	-3.859	-3.314
SD	3.318	2.584	2.441	1.492	3.410	2.608

2. Supplementary Information to the Results Section: Post-hoc Comparisons of Significant Interactions

Below we present supplementary tables to provide complete information on the post-hoc comparisons of significant interactions.

Supplementary Table 2. Analysis of Evaluation Scores for "Directional Cues versus Non-predictive Cues"; Post-Hoc Comparisons with Bonferroni Correction on the Interaction between Image Content and Cue Type.

		Mean Difference	SE	t	p_{bonf}
Immoral, Directional	Neutral, Directional	3.521	0.247	14.254	< .001
	Moral, Directional	0.369	0.247	1.495	1.000
	Immoral, Non-predictive	0.579	0.158	3.662	0.007
	Neutral, Non-predictive	3.749	0.264	14.226	< .001
	Moral, Non-predictive	1.032	0.264	3.917	0.003
Neutral, Directional	Moral, Directional	-3.151	0.247	-12.759	< .001
	Immoral, Non-predictive	-2.942	0.264	-11.164	< .001
	Neutral, Non-predictive	0.228	0.158	1.446	1.000
	Moral, Non-predictive	-2.488	0.264	-9.443	< .001
Moral, Directional	Immoral, Non-predictive	0.209	0.264	0.794	1.000
	Neutral, Non-predictive	3.380	0.264	12.825	< .001
	Moral, Non-predictive	0.663	0.158	4.195	0.001
Immoral, Non-predictive	Neutral, Non-predictive	3.170	0.247	12.836	< .001
	Moral, Non-predictive	0.454	0.247	1.836	1.000
Neutral, Non-predictive	Moral, Non-predictive	-2.717	0.247	-11.000	< .001

Note. P-value adjusted for comparing a family of 15

Note. Results are averaged over the levels of: Volatility

Supplementary Table 3. Analysis of Evaluation Scores for "Directional Cues versus Non-predictive Cues"; Post-Hoc Comparisons with Bonferroni Correction on the Interaction between Image Content and Volatility.

		Mean Difference	SE	t	p_{bonf}
Immoral, HSD	Neutral, HSD	3.602	0.243	14.834	< .001
	Moral, HSD	0.761	0.243	3.132	0.038
	Immoral, LSD	0.511	0.113	4.509	< .001
	Neutral, LSD	3.600	0.244	14.775	< .001
	Moral, LSD	0.573	0.244	2.353	0.320
Neutral, HSD	Moral, HSD	-2.842	0.243	-11.702	< .001
	Immoral, LSD	-3.091	0.244	-12.687	< .001
	Neutral, LSD	-0.002	0.113	-0.020	1.000
	Moral, LSD	-3.029	0.244	-12.431	< .001
Moral, HSD	Immoral, LSD	-0.249	0.244	-1.024	1.000
	Neutral, LSD	2.839	0.244	11.654	< .001
	Moral, LSD	-0.187	0.113	-1.651	1.000
Immoral, LSD	Neutral, LSD	3.089	0.243	12.720	< .001
	Moral, LSD	0.062	0.243	0.256	1.000
Neutral, LSD	Moral, LSD	-3.027	0.243	-12.463	< .001

Note. P-value adjusted for comparing a family of 15

Note. Results are averaged over the levels of: Cue Type

Supplementary Table 4. Analysis of Evaluation Scores for "Directional Cues versus Non-predictive Cues"; Post-Hoc Comparisons with Bonferroni Correction on the Interaction between Volatility and Cue Type.

		Mean Difference	SE	t	p_{bonf}
HSD, Directional	LSD, Directional	0.247	0.081	3.054	0.022
	HSD, Non-predictive	0.630	0.126	4.987	< .001
	LSD, Non-predictive	0.597	0.136	4.391	< .001
LSD, Directional	HSD, Non-predictive	0.383	0.136	2.814	0.043
	LSD, Non-predictive	0.350	0.126	2.771	0.052
HSD, Non-predictive	LSD, Non-predictive	-0.033	0.081	-0.405	1.000

Note. P-value adjusted for comparing a family of 6

Note. Results are averaged over the levels of: Image Content

Supplementary Table 5. Analysis of Response Times for "Directional Cues versus Non-predictive Cues"; Post-Hoc Comparisons with Bonferroni Correction on the Interaction between Image Content and Cue Type.

		Mean Difference	SE	t	p_{bonf}
Immoral, Directional	Neutral, Directional	-0.261	0.048	-5.468	< .001
	Moral, Directional	0.039	0.048	0.825	1.000
	Immoral, Unpredictive	0.015	0.044	0.335	1.000
	Neutral, Unpredictive	-0.241	0.052	-4.639	< .001
	Moral, Unpredictive	-0.065	0.052	-1.243	1.000
Neutral, Directional	Moral, Directional	0.301	0.048	6.293	< .001
	Immoral, Unpredictive	0.276	0.052	5.313	< .001
	Neutral, Unpredictive	0.020	0.044	0.456	1.000
	Moral, Unpredictive	0.197	0.052	3.784	0.004
Moral, Directional	Immoral, Unpredictive	-0.025	0.052	-0.473	1.000
	Neutral, Unpredictive	-0.280	0.052	-5.398	< .001
	Moral, Unpredictive	-0.104	0.044	-2.349	0.320
Immoral, Unpredictive	Neutral, Unpredictive	-0.256	0.048	-5.357	< .001
	Moral, Unpredictive	-0.079	0.048	-1.664	1.000
Neutral, Unpredictive	Moral, Unpredictive	0.176	0.048	3.693	0.005

Note. P-value adjusted for comparing a family of 15

Note. Results are averaged over the levels of: Volatility

Supplementary Table 6. Analysis of Response Times for "Directional Cues versus Non-predictive Cues"; Post-Hoc Comparisons with Bonferroni Correction on the Interaction between Image Content and Volatility.

		Mean Difference	SE	t	p_{bonf}
Immoral, HSD	Neutral, HSD	-0.341	0.050	-6.859	< .001
	Moral, HSD	-0.043	0.050	-0.859	1.000
	Immoral, LSD	-0.043	0.039	-1.126	1.000
	Neutral, LSD	-0.220	0.045	-4.868	< .001
	Moral, LSD	-0.041	0.045	-0.904	1.000
Neutral, HSD	Moral, HSD	0.298	0.050	6.000	< .001
	Immoral, LSD	0.297	0.045	6.574	< .001
	Neutral, LSD	0.121	0.039	3.125	0.038
	Moral, LSD	0.300	0.045	6.632	< .001
Moral, HSD	Immoral, LSD	-8.013×10^{-4}	0.045	-0.018	1.000
	Neutral, LSD	-0.177	0.045	-3.925	0.002
	Moral, LSD	0.002	0.039	0.047	1.000
Immoral, LSD	Neutral, LSD	-0.177	0.050	-3.556	0.008
	Moral, LSD	0.003	0.050	0.052	1.000
Neutral, LSD	Moral, LSD	0.179	0.050	3.609	0.007

Note. P-value adjusted for comparing a family of 15

Note. Results are averaged over the levels of: Cue Type
